# Effectiveness of the clinical decision support tool ESR eGUIDE for teaching medical students the appropriate selection of imaging tests: randomized cross-over evaluation

**DOI:** 10.1007/s00330-020-06942-2

**Published:** 2020-05-20

**Authors:** Torsten Diekhoff, Franz Kainberger, Laura Oleaga, Marc Dewey, Elke Zimmermann

**Affiliations:** Department of Radiology, Charité – Universitätsmedizin Berlin, Humboldt-Universität zu Berlin, Freie Universitat Berlin, Charitéplatz 1, 10117 Berlin, Germany

**Keywords:** Random allocation, Clinical decision support systems, Diagnostic imaging, Learning, Medical students

## Abstract

**Objectives:**

To evaluate ESR eGUIDE—the European Society of Radiology (ESR) e-Learning tool for appropriate use of diagnostic imaging modalities—for learning purposes in different clinical scenarios.

**Methods:**

This anonymized evaluation was performed after approval of ESR Education on Demand leadership. Forty clinical scenarios were developed in which at least one imaging modality was clinically most appropriate, and the scenarios were divided into sets 1 and 2. These sets were provided to medical students randomly assigned to group A or B to select the most appropriate imaging test for each scenario. Statistical comparisons were made within and across groups.

**Results:**

Overall, 40 medical students participated, and 31 medical students (78%) answered both sets. The number of correctly chosen imaging methods per set in these 31 paired samples was significantly higher when answered with versus without use of ESR eGUIDE (13.7 ± 2.6 questions vs. 12.1 ± 3.2, *p* = 0.012). Among the students in group A, who first answered set 1 without ESR eGUIDE (11.1 ± 3.2), there was significant improvement when set 2 was answered with ESR eGUIDE (14.3 ± 2.5, *p* = 0.013). The number of correct answers in group B did not drop when set 2 was answered without ESR eGUIDE (12.4 ± 2.6) after having answered set 1 first with ESR eGUIDE (13.0 ± 2.7, *p* = 0.66).

**Conclusion:**

The clinical decision support tool ESR eGUIDE is suitable for training medical students in choosing the best radiological imaging modality in typical scenarios, and its use in teaching radiology can thus be recommended.

**Key Points:**

*• ESR eGUIDE improved the number of appropriately selected imaging modalities among medical students.*

*• This improvement was also seen in the group of students which first selected imaging tests without ESR eGUIDE.*

*• In the student group which used ESR eGUIDE first, appropriate selection remained stable even without the teaching tool.*

**Electronic supplementary material:**

The online version of this article (10.1007/s00330-020-06942-2) contains supplementary material, which is available to authorized users.

## Introduction

The Choosing Wisely initiative has identified certain procedures that are not recommended due to low evidence or because they can even be harmful [1, 2]. However, the initiative’s lists were also influenced by political and economic aspects and may thus have limited uptake and influence [3]. Therefore, appropriate-use criteria, ideally developed using evidence-based [4] and Delphi processes [5], hold the greatest potential to address geographical inconsistencies in clinical imaging indications and to reduce the inappropriate conduct of imaging tests while ensuring that necessary imaging tests are done for the right patient at the right point in time [6].

Teaching medical students about the appropriate use of imaging tests could become a key factor in avoiding low-value health care and overutilization of diagnostic imaging [7–9]. Especially e-Learning might be suited for interactively teaching medical students the true sense of clinical decision support systems [10] including the Bayesian perspective to diagnostic test selection [11, 12]. The European Society of Radiology (ESR) has developed ESR iGUIDE as a clinical decision support system for appropriate imaging test selection in a variety of clinical scenarios [13]. Recently, this initiative, which became available in 2018, was extended to the teaching of medical students and continuous medical education, and, under the name ESR eGUIDE, was made available as an online electronic learning (e-Learning) source. However, its capability to guide the decision for the appropriate imaging test in a given clinical scenario and to educate medical students have not yet been analyzed.

Therefore, the aim of our study was to test the effectiveness of this e-Learning tool as a clinical decision support in teaching medical students. We hypothesized that ESR eGUIDE might improve the appropriateness of imaging test selection by medical students and thus set up a random cross-over design for evaluation of medical students to test this hypothesis in a variety of clinical scenarios.

## Materials and methods

### Study design

**Fig. 1:**
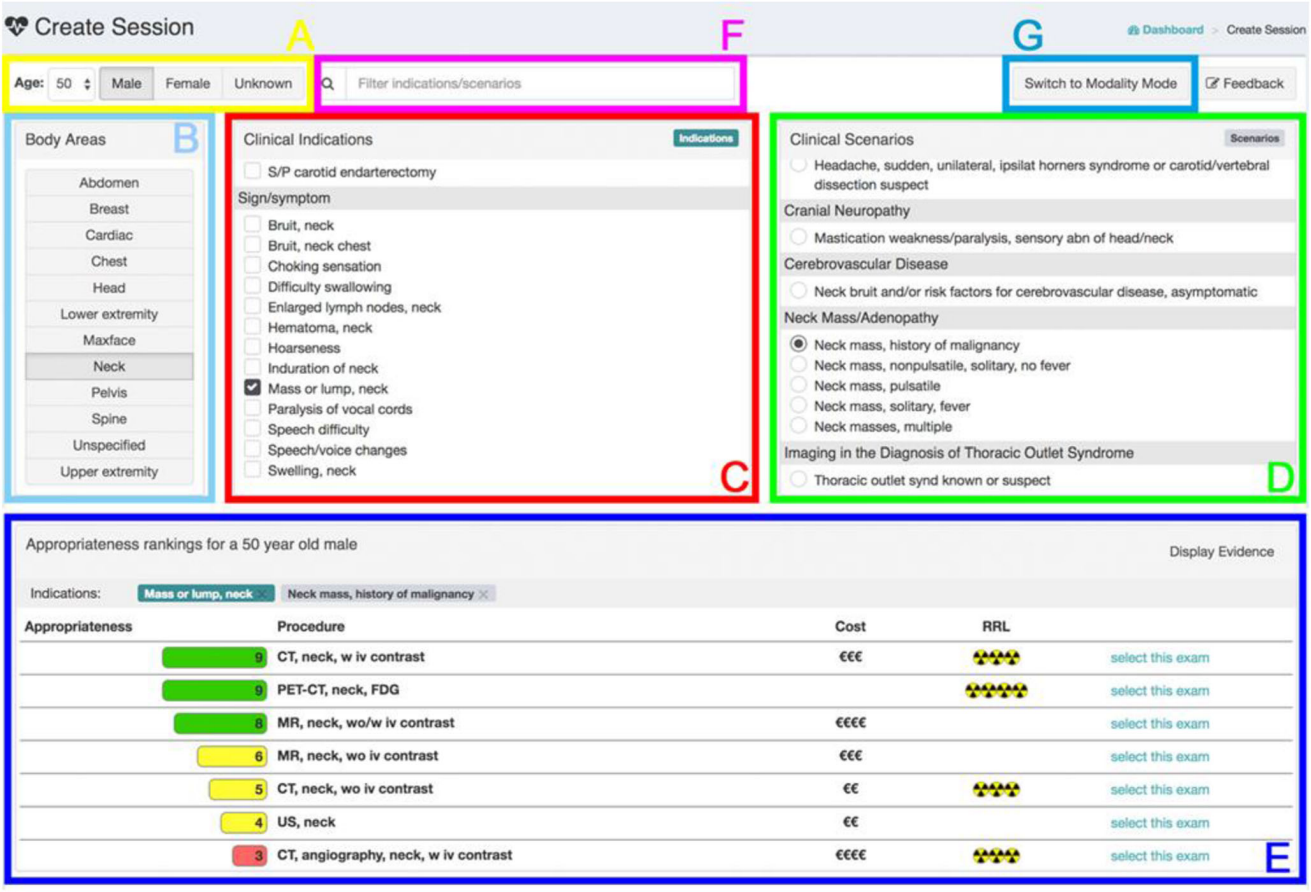
Overview of the ESR eGUIDE platform. (A) The first step is choosing the right age and gender of the patient. (B) The second step is choosing the anatomic region. It is possible to select multiple indications. (C) The third step, clinical indication, e.g., neck mass. (D) Choose clinical scenario, e.g., history of malignancy (optional). (E) Review the results with appropriateness score, costs, and radiation indicator for each procedure. (F) You might also use the quick search. (G) You can also search for a specific modality for your clinical scenario

This anonymized study was performed at a single university hospital in Europe (Charité). The study was approved by ESR leadership of the Education on Demand e-Learning group. The study took place as a random cross-over evaluation of medical students in 2017 and 2018 and utilized the first version of the ESR eGUIDE platform (Fig. 1), which transitioned into a new platform at the turn of the year 2018. The ESR eGUIDE allows to review the evidence-based appropriateness of different modalities, and their radiation exposure and costs. The appropriateness is indicated by a score on a 1 to 9 scale (1–3: usually not appropriate, 4–6: may be appropriate, 7–9: usually appropriate) together with an indicator of radiation exposure (0 to 5, 0: no radiation, 5: 10–30 mSv radiation exposure) and costs of the examination.

### Clinical scenarios and test sets

Forty clinical scenarios were developed by a core team at Charité. In each scenario, at least one imaging modality was clinically most appropriate: angiography, computed tomography, magnetic resonance imaging, x-ray, or sonography. These 40 scenarios were divided into set 1 and set 2, each including 20 clinical scenarios, and are provided in the Electronic Supplementary Material.

### Study conduct

The two sets of 20 clinical scenarios each were provided to medical students randomly assigned to group A or B to select the most appropriate imaging test for each scenario. Medical student group A first answered set 1 of 20 clinical scenarios without ESR eGUIDE, followed by set 2 with ESR eGUIDE. Medical student group B first answered set 1 of 20 clinical scenarios with ESR eGUIDE, followed by set 2 without ESR eGUIDE.

### Study administration

The purpose of the study was introduced to medical students participating in a voluntary radiology lecture on playful learning imaging test selection (“when and which imaging test to select”) and which also explained general principles of the five diagnostic imaging tests. Moreover, the Bayes principle including pre-test probability estimation for test selection was discussed with a handful of clinical examples which were different from those included in the two sets of 20 clinical scenarios. Following this lecture, medical students could enroll in an e-Learning activity which formed this random cross-over evaluation study. Enrolled students were randomly assigned to medical student group A or B and received access codes for ESR eGUIDE. Following this registration, students could go through the 40 clinical scenarios included in the two test sets. Students did not know before study conduct to which of the two groups they were assigned and could withdraw from participation at any time. At the end of having answered all scenarios, participants received an analysis of their performance in appropriate diagnostic imaging test selection.

### Statistical analysis

For the prevalence of correct responses, descriptive statistics were used and comparisons were performed using a two-tailed Wilcoxon matched-pairs signed rank. Statistical comparisons were made within and across groups considering a *p* value below 0.05 as statistically significant. We performed all analysis with the statistical software Prism (Version 8.1; GraphPad).

## Results

### Participation

**Fig. 2:**
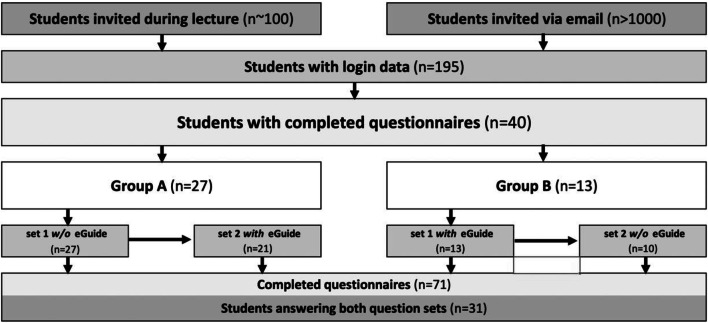
Participation flow chart. Students were invited using two ways: during a lecture and via e-mail (note that lecture participants may also have received an e-mail as lecture participation is not tracked individually). Overall, 195 students requested login data for ESR eGUIDE. Of those requesting to participate 40 completed questionnaires. Students were randomly assigned to two groups (A and B). Group A completed set 1 of clinical scenarios first without ESR eGUIDE and then set 2 with ESR eGUIDE and group B completed first set 1 with ESR eGUIDE followed by set 2 without ESR eGUIDE. Over 1000 students were invited to participate. Nearly 200 students requested and were granted login data. Forty students completed questionnaires. However, only 31 completed both question sets.

The number of students invited and those receiving login data is shown in Fig. 2. Overall, 40 medical students participated in this random evaluation of the ESR eGUIDE teaching tool for appropriate medical imaging test selection. Thirty-one of the 40 medical students (78%) answered both sets of clinical scenarios (Fig. 2).

### Appropriate selection of imaging tests with and without ESR eGUIDE

**Fig. 3:**
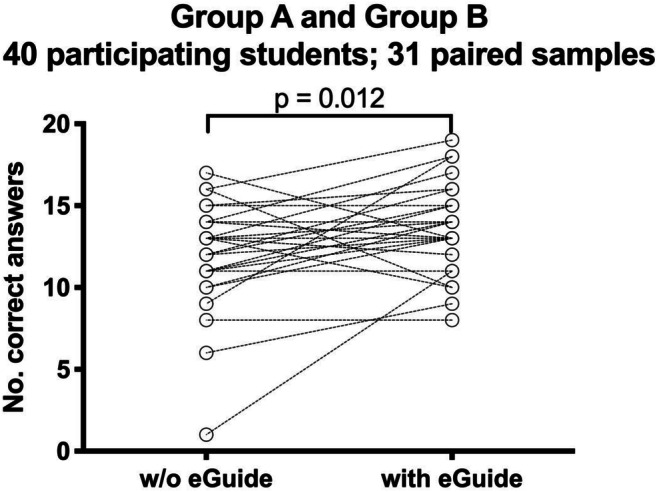
Significant improvement in questionnaire value (min 0, max 20) in the 31 paired samples through the use of ESR eGUIDE (*p* = 0.012)

**Fig. 4:**
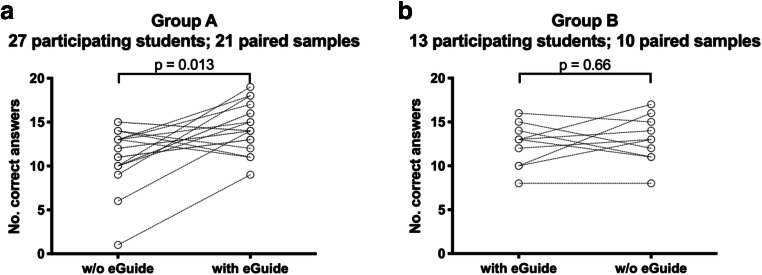
**a**, **b** Change in questionnaire values in group A (w/o ESR eGUIDE first) and group B (with eGIUDE first)

The number of appropriately chosen imaging methods per set (mean ± SD) in the 31 paired samples of the medical student answering both sets of clinical scenarios was significantly higher when answered with (13.7 ± 2.6) as compared to without (12.1 ± 3.2) ESR eGUIDE (*p* = 0.012, Fig. 3). Among the students in group A, who answered set 1 without ESR eGUIDE first (11.1 ± 3.2), there was a significant improvement when set 2 was answered with ESR eGUIDE (14.3 ± 2.5, *p* = 0.013, Fig. 4a). For both tests, the elimination of one outlier did not change the results significantly (*p* = 0.022 and *p* = 0.002, respectively). The number of correct answers in group B did not decline when set 2 was answered without ESR eGUIDE (12.4 ± 2.6) after having answered set 1 first with ESR eGUIDE (13.0 ± 2.7, *p* = 0.66, Fig. 4b).

## Discussion

This random cross-over evaluation was conducted to test the effectiveness of ESR eGUIDE to improve the appropriate selection of diagnostic imaging tests by medical students in different clinical scenarios. We found that ESR eGUIDE significantly improved the proportion of appropriately selected imaging tests. This was most likely a result of the better guidance provided by listings of appropriate, indeterminate, and inappropriate imaging tests for certain scenarios. These results are of relevance as they may inform medical training curricula, such as the ESR Curriculum for Undergraduate Radiological Education (https://www.myesr.org/media/229), by putting greater weight on e-Learning and radiology in undergraduate teaching [14–16].

We think that this survey has two implications. Firstly, clinical decision support tools are suitable to improve students’ knowledge in selecting appropriate diagnostic imaging tests. This may help in disseminating ESR eGUIDE to other institutions using the evidence generated in this study. Secondly, in the student group which used ESR eGUIDE first, similarly high appropriate test ordering was found when this group analyzed the second set of clinical scenarios without ESR eGUIDE. This suggests that sustained skills might develop with the use of ESR eGUIDE and persist even in future settings when clinical decision support systems [17, 18] are not at hand anymore.

Digital transformation will also fundamentally change e-Learning to reach new levels using image interaction possibilities, which were not included in the present study, yet may further increase radiology knowledge and skills of medical students [19]. Interestingly, e-Learning can also be used to increase skills in optimizing acquisition and reducing artifacts in magnetic resonance imaging [20]. For a long time, e-Learning has already been used to assist medical students in understanding anatomy [21]. Including adaptive tutorials in teaching diagnostic test selection principles might further improve the understanding of imaging test properties by medical students. This was not yet included in the present analysis due to the complexity of setting up personalized learning experiences [22]. Whether or not additional teaching of students by radiology residents using web-based educational material may improve clinical decision-making skills is also not clear yet.

In a survey, medical students participating in a radiology clerkship stated that they would be interested in using the appropriateness criteria of the American College of Radiology (ACR) for effective utilization of imaging tests and evidence-based imaging [23]. Another study among third-year medical students, however, showed that use of the ACR appropriate-use criteria was actually low and did not increase after interventions improving familiarity with such criteria [24]. The most important reason for this failure was the lack of a quick, easy-to-use online mobile application–based interface [24]. We tried to tackle this barrier by evaluating ESR eGUIDE as an online interactive teaching interface for the appropriate utilization of diagnostic imaging tests.

There is an increasing clinical demand for better training of students in selecting the most appropriate imaging test for the right patients at the right point in time, e.g., using ESR eGUIDE. e-Learning approaches to teaching might be best suited for individually adjusting the learning experience. As a result, students might be put in a position to provide greater value to patient care through more consistent and evidence-based selection of imaging tests that are best suited to individual clinical scenarios [25, 26].

This study is limited by its single-center design and the small number of participating medical students. Especially for the comparisons within group B, the statistics might be underpowered—it might be that no significant differences were found due to the small sample size of 13 participants. Only a fraction of the more than 1000 invited students applied for log-in data (195) and only a fraction of those completed one (40) or both (31) questionnaires. However, we have no information about those who participated nor why the others have decided to not respond to the invitation or to not complete the clinical scenarios. Moreover, it is likely that the participants are more committed than students not participating, which might have biased our results. The random evaluation and cross-over design are advantages that allow balanced comparisons of the effectiveness of ESR eGUIDE. Larger studies in more than one medical university are recommended and appear worthwhile based on the initial favorable results obtained here.

In summary, this random evaluation shows that guiding medical students towards appropriate imaging test selection results in significantly more correctly indicated tests being selected for the individual case scenarios.

## Electronic supplementary material

The two sets of clinical scenarios (each 20) can be found in the Electronic Supplementary Material.

ESM 1 (DOCX 29 kb)

## Abbreviations

ACR: American College of Radiology
ESR: European Society of Radiology
