# Supplementary material for: Effectiveness of the clinical decision support tool ESR eGUIDE for teaching medical students the appropriate selection of imaging tests: randomized cross-over evaluation
Source: Eur Radiol. 2020 May 20;30(10):5684–9. doi: 10.1007/s00330-020-06942-2 (PMC7476994; doi:10.1007/s00330-020-06942-2)
Supplement: Supplementary file 1 — (DOCX 29 kb) [file 330_2020_6942_MOESM1_ESM.docx]

**ESM** Forty clinical scenarios

A core team at Charité developed 40 scenarios in which at least one of the following imaging modalities was clinically most appropriate: angiography, computed tomography, magnetic resonance imaging, x-ray, or sonography. These 40 scenarios were divided into set 1 and set 2, each including 20 scenarios that were presented in random order.

Answers:

A: Angiography

B: Computed tomography

C: Magnetic resonance imaging

D: X-rax

E: Sonography

Clinical Scenario Set 1

1:

A 48-year-old male patient walks into the emergency department with pain in the right lower abdomen. The patient states that the pain has persisted for three days and that he has never experienced such severe pain before. No fever. The right lower quadrant is tense. Lab tests: elevated inflammatory markers. You suspect acute appendicitis.

Question: Which imaging modality is most appropriate here?

Correct answer: E

2:

A 63-year-old female patient is brought to the emergency department by ambulance. Her personality is markedly altered, and her responsiveness deteriorating (probably since the day before). A history cannot be obtained from her. Her husband reports a history of melanoma on the back first diagnosed three years earlier. Normal follow-up by GP. You suspect cerebral metastasis possibly with hemorrhage or entrapment.

Question: Which imaging modality is most appropriate here?

Correct answer: B

3:

A 65-year-old male patient presents with acute onset of abdominal pain extending into the back. He reports a history of arterial hypertension and vascular sclerosis. An external ultrasound examination suggests widening of the abdominal aorta. You suspect aneurysm rupture.

Question: Which imaging modality is most appropriate here?

Correct answer: B

4:

A 35-year-old female patient presents with severe, colicky flank pain on the left. She reports having vomited once. No fever. Normal passage of urine and normal bowel movements. Urinary Stix test: erythrocytes +++. You suspect a renal calculus.

Question: Which imaging modality is most appropriate here?

Correct answer: B, D

5:

A 65-year-old male patient with a history of liver cirrhosis presents with a sensation of pressure in the right upper abdomen. Lab findings include elevated alpha-fetoprotein. You suspect hepatocellular carcinoma.

Question: Which imaging modality is most appropriate here?

Correct answer: C

6:

A 68-year-old obese patient presents to the emergency department with typical angina pectoris, vertigo, and nausea. He has a history of type II diabetes mellitus and arterial hypertension. The ECG shows no ST segment changes and sinus rhythm. Lab tests: elevated CK-MB and troponin. You suspect acute coronary artery occlusion.

Question: Which imaging modality is most appropriate here?

Correct answer: A

7:

A 75-year-old female patient presents to the emergency department with acute dyspnea. Rales over both lungs on auscultation with a dull percussion note over both lower pulmonary lobes. No cough, no fever, no expectoration. You suspect cardiac decompensation.

Question: Which imaging modality is most appropriate here?

Correct answer: D

8:

A 40-year-old bicyclist is brought in by an emergency physician. He fell over the handlebar of his bike trying to avoid a collision with a pedestrian suddenly appearing in front of him. Abrasive wound of left lower arm, hematoma in left flank. Diffuse abdominal pain. The patient is alert, oriented, and complains of nausea (having already vomited once), and diffuse abdominal pain. You wish to perform an exploratory test to rapidly find out whether there is free fluid/blood in the abdomen.

Question: Which imaging modality is most appropriate here?

Correct answer: E

9:

An 83-year-old male patient living in a nursing home became unresponsive in the morning (first episode). Drooping left mouth corner and slurred speech. You suspect an ischemic stroke and intend to initiate thrombolytic treatment.

Question: Which imaging modality is most appropriate here?

Correct answer: B

10:

A 44-year-old man presents after falling on the back from a height of about 2 m from a ladder. The patient reports back pain and is not able to move his legs adequately upon request. There are contusion marks on the back. You suspect a fracture with damage to nerve structures.

Question: Which imaging modality is most appropriate here?

Correct answer: B, C

11:

A 54-year-old male patient presents with an acute new swelling submentally on the left. The mass is palpable subcutaneously, tender, and mobile. The overlying skin is slightly red and livid. No fever. Elevated inflammatory parameters. You suspect an abscess.

Question: Which imaging modality is most appropriate here?

Correct answer: B, E

12:

A 46-year-old female patient presents for aftercare with a history of breast cancer treated by breast-sparing surgery 2 years earlier. Nonirritable scar clinically. Ultrasound provides no suspicious findings, no lymphadenopathy. The patient reports no complaints.

Question: Which imaging modality is most appropriate here?

Correct answer: D

13:

A 4-year-old boy is presented after a fall from a jungle gym. He has severe pain in the left lower arm. You wish to rule out a fracture.

Question: Which imaging modality is most appropriate here?

Correct answer: D

14:

A 64-year-old female patient presents with new-onset back pain. No trauma. Four-year history of breast cancer. An initial X-ray shows no acute vertebral fractures but a patchy appearance of spinal bone structures. You wish to rule out spinal metastasis.

Question: Which imaging modality is most appropriate here?

Correct answer: C

15:

A 57-year-old male patient presents with upper abdominal pain extending in a belt-like fashion. Elevated inflammatory parameters and serum lipase. You suspect pancreatitis.

Question: Which imaging modality is most appropriate here?

Correct answer: B, E

16:

A 13-year-old girl presents with load-dependent pain of the left knee. No adequate trauma. Pain occurs after exercise. No relevant joint effusion. Conventional radiograph shows altered bone structure of periarticular femur condyle. You suspect dissecting osteochondrosis.

Question: Which imaging modality is most appropriate here?

Correct answer: C

17:

A 47-year-old male patient (tiler) presents with severe knee pain. No adequate trauma. Pain is fairly persistent and not load-dependent. Based on the clinical examination, you suspect a medial meniscal lesion.

Question: Which imaging modality is most appropriate here?

Correct answer: C

18:

A 32-year-old female patient presents with pain of the right calf 10 days after an arthroscopy. The clinical examination reveals a slight increase in circumference on the right. You suspect deep vein thrombosis of the leg.

Question: Which imaging modality is most appropriate here?

Correct answer: E

19:

A 73-year-old male patient fell on his hand, slipping on wet leaves while walking. The left hand is very painful and cannot be moved adequately. You suspect a distal radial fracture.

Question: Which imaging modality is most appropriate here?

Correct answer: D

20:

A 42-year-old male patient presents with acute massive abdominal pain in all four quadrants. The abdomen is rigid. Marked tension. History of laparoscopy 12 years earlier. You suspect an ileus.

Question: Which imaging modality is most appropriate here?

Correct answer: B

Clinical Scenario Set 2

1:

Case vignette: A 56-year-old female patients presents to the emergency department with severe right-sided pain of the upper abdomen. The clinical examination reveals a tender right upper quadrant with a positive Murphy sign. The patient’s CRP is elevated with borderline elevation of leukocytes and elevated cholestase parameters. You suspect cholecystitis.

Question: Which imaging modality is most appropriate here?

Correct answer: E

2:

Case vignette: A 28-year-old patient is hospitalized in an infectious diseases ward for atypical pneumonia developing on the basis of a long-standing HIV infection. Three days after initiation of treatment, he reports severe headache, double vision, and nausea. The neurologic examination reveals left hemiparesis mostly affecting the brachiofacial region. You suspect a brain abscess.

Question: Which imaging modality is most appropriate here?

Correct answer: C

3:

A 78-year-old female patient with most severe back pain is brought in by an emergency physician. She describes her sensation as if someone is stabbing a knife into her back. The initial examination reveals tachycardia with a systolic blood pressure difference between both arms of over 30 mmHg. The D-dimer level is elevated. You suspect thoracic aortic dissection.

Question: Which imaging modality is most appropriate here?

Correct answer: B

4:

A 42-year-old male patient presents to the emergency department with severe flank pain. The abdomen is soft but tender on the left side. Exploratory blood tests reveal no abnormalities. The urinary Stix test shows elevated erythrocytes. Initial ultrasound reveals first-degree urinary obstruction on the left. You suspect a ureteral stone.

Question: Which imaging modality is most appropriate here?

Correct answer: B

5:

A 55-year-old female patient with a history of breast-sparing surgery for breast cancer shows unclear liver lesions in an initial routine ultrasound examination. Blood work is normal. You wish to differentiate liver metastasis from benign lesions.

Question: Which imaging modality is most appropriate here?

Correct answer: C

6:

A 75-year-old female patient with dyspnea and chest pain is brought in by an emergency physician. She recalls no acute trauma but reports having stumbled while on a bus trip through Italy 2 days earlier. Since then there has been swelling of her left leg. Saturation is 85% under ambient conditions. D-dimers are elevated. You suspect pulmonary artery embolism.

Question: Which imaging modality is most appropriate here?

Correct answer: B

7:

A 78-year-old male patient presents to the emergency department with acute exacerbation of COPD. He reports progressive cough with yellowish-green sputum having persisted for two days. Saturation is 92% under ambient conditions. Blood tests reveal elevated CRP and leukocytes. You suspect pneumonia.

Question: Which imaging modality is most appropriate here?

Correct answer: D

8:

A 32-year-old male patient presents to the emergency department with very severe paroxysmal headache. He reports having never had such severe headache before. The headache occurred out of the blue and did not respond to ibuprofen. He recalls no trauma. Blood work is normal. You suspect subarachnoid bleeding.

Question: Which imaging modality is most appropriate here?

Correct answer: B, C

9:

A 67-year-old female patient is brought in by an emergency physician, who was called to a concert hall and found her still responsive when he arrived on the scene. Now she is comatose. The emergency physician’s initial examination revealed right-sided sensorimotor hemiparesis. You suspect hyertensive mass bleeding.

Question: Which imaging modality is most appropriate here?

Correct answer: B

10:

A 39-year-old male patient reports a two-week history of back pain extending into the right leg down into the foot. The onset was sudden and responded only little to nonsteroidal antirheumatic medication. The clinical examination reveals mildly disturbed sensibility and weakness of dorsiflection of the foot. You suspect a vertebral disk prolapse.

Question: Which imaging modality is most appropriate here?

Correct answer: C

11:

A 23-year-old woman presents to the emergency department with new-onset, painless hematuria. She recalls no trauma. Lab tests are normal. You suspect hemorrhage into a renal cyst.

Question: Which imaging modality is most appropriate here?

Correct answer: E

12:

A 55-year-old woman presents for breast cancer screening. She reports no complaints. Her last lab tests performed by her GP revealed no abnormalities.

Question: Which imaging modality is most appropriate here?

Correct answer: D

13:

A young woman takes her 3- and half-year-old boy to the hospital’s emergency department. She reports that he has complained of headache and nausea with repeated vomiting for about seven hours. Upon questioning, she reports that the boy had fell on his head from a height of about 1.5 m in the playground on the day before. After the fall, he was not responsive for a short moment. You suspect intracranial bleeding.

Question: Which imaging modality is most appropriate here?

Correct answer: C

14:

A 74-year-old male patient presents with progressive back pain. He had prostatectomy for prostate cancer two years earlier. The lab examination reveals elevated prostate-specific antigen. You suspect bone metastasis.

Question: Which imaging modality is most appropriate here?

Correct answer: C

15:

A 65-year-old female patient presents with marked, left-sided pain of the lower abdomen with slow progression over the last 24 hours. She also reports fever and chills. The clinical examination reveals marked tenderness in the left lower quadrant. You suspect diverticulitis.

Question: Which imaging modality is most appropriate here?

Correct answer: B

16:

An 8-year-old boy is presented to the orthopedic service with hip pain. Internal rotation and abduction are limited by pain. You suspect aseptic bone necrosis or Perthes disease.

Question: Which imaging modality is most appropriate here?

Correct answer: C, D

17:

A 32-year-old patient presents with a status after knee distortion during her skiing vacation. An x-ray performed after the accident was interpreted to be normal. The Lachmann and pivot-shift tests are positive. You suspect rupture of the anterior cruciate ligament.

Question: Which imaging modality is most appropriate here?

Correct answer: C

18:

A 52-year-old male patient presents with acute pain of the right leg. The leg is cold. He reports that the pain occurred out of the blue 2 hours earlier. The skin is marbled with partially livid discoloration. Groin pulses are obtainable while popliteal and foot pulses are absent. You suspect acute vascular occlusion.

Question: Which imaging modality is most appropriate here?

Correct answer: A

19:

A 20-year-old female patient presents with persisting pain following ankle joint distortion one day earlier. She reports problems running. The joint is swollen and tender, especially above the lateral malleolus muscle. Further clinical evaluation is precluded by pain. You suspect a fracture.

Question: Which imaging modality is most appropriate here?

Correct answer: D

20:

A 67-year-old male patient with known coronary heart disease presents to the emergency department with massive progressive abdominal pain of sudden onset about 5 hours earlier. The entire abdomen is painful on pressure and very tender. Inflammatory parameters are slightly elevated, while lactate is markedly elevated. You suspect intestinal ischemia.

Question: Which imaging modality is most appropriate here?

Correct answer: B
